# Anorexia nervosa in the times of COVID-19 pandemic is it different than before?

**DOI:** 10.1192/j.eurpsy.2023.917

**Published:** 2023-07-19

**Authors:** H. Y. Yılancıoğlu, S. H. Tokmak, B. Yuluğ Taş, D. Çek, B. Özbaran

**Affiliations:** Child and Adolescent Psychiatry, Ege University Hospital, Izmir, Türkiye

## Abstract

**Introduction:**

The COVID-19 pandemic control measures such as isolation and social restrictions are related to an increase in the incidence of anorexia nervosa and deteriorating symptoms by increased social media exposure, limited access to psychiatric services, disruptions in relationships between families and adolescents.

**Objectives:**

Aim of study was to investigate the psychiatric and psychosocial impacts and clinical changes in anorexia nervosa patients, who applied to the Ege University Child and Adolescent Psychiatry for the first time in 2018, during the 2019-2022 pandemic period.

**Methods:**

Our study was carried out 35 anorexia nervosa patients. Voluntary written informed consent, self-report form; using The Visual Analog Scale (VAS), Screen for Child Anxiety Related Disorders Scale (SCARED), Eating Attitudes Test (EAT), The Quality of Life Scale (QOLS), The Difficulties in Emotion Regulation Scale (DERS), The Autism Spectrum Screening Questionnaire (ASSQ), Atilla Turgay DSM-4 Based Screening and Evaluation Scale for Behavioral Disorders in Children and Adolescents (TURGAY) forms filled out online. Clinical diagnosis and progress are obtained through archieve records by The Kiddie Schedule for Affective Disorders and Schizophrenia (K-SADS) and Clinical Global Impression (CGI) scales. Approval number is 22-6T/7, Ege University Ethics Committee.

**Results:**

In 35 patients;15 female patients completed all the forms. The mean age was 16.67±1.63 years. 11 (73.33%) patients have at least one comorbidity; 7 (46.66%) patients have major depressive disorder, 3 (20.00%) anxiety disorder, 2 (13.33%) attention deficit and hyperactivity disorder, 1 (6.66%) mood disorder. The SCARED score was 37.23±12.67, and the CDI score was 17.23±10.85. When comparing the pre-pandemic period, obsession level (z=-2.254, p=.024), exercise level (z=-2.508, p=.012), technology exposure (z=-2.290, p=.022) is increased; level of social activity (z=-2.206, p=.027), the quality of education (z=-2.167, p=.030), and the perception of learning (z=-3.301, p=.008) decreased during pandemic. Quality of life scores was inversely correlated with eating attitudes scores (r=-.601, p=.039). It was noteworthy that number of admissions from the first appointments was higher in participants, compared to the patients who did not participate in the study (n=20) (p=.033). The first admission BMI values were negatively correlated with CGI scores of the patients (r=-.743, p=.002).

**Conclusions:**

As a result, Covid-19 has negative psychosocial effects in anorexia nervosa symptoms such as increased excercise at home and technology exposure; decreased in social activity. Sharing clinical experiences about our patients’ mental health may be beneficial in planning the treatment processes and approach for further unexpected extraordinary situations.

**Disclosure of Interest:**

None Declared

